# Let's Face It—Complex Traits Are Just Not That Simple

**DOI:** 10.1371/journal.pgen.1004724

**Published:** 2014-11-06

**Authors:** Benedikt Hallgrimsson, Washington Mio, Ralph S. Marcucio, Richard Spritz

**Affiliations:** 1 Department of Cell Biology and Anatomy and the Alberta Children's Hospital Research Institute, University of Calgary, Calgary, Alberta, Canada; 2 Department of Mathematics, Florida State University, Tallahassee, Florida, United States of America; 3 Department of Orthopedics, University of California, San Francisco, San Francisco, California, United States of America; 4 Department of Pediatrics and Human Medical Genetics and Genomics Program, University of Colorado School of Medicine, Denver, Colorado, United States of America; Seattle Children's Research Institute, United States of America

The idea that we can reconstruct a human face from a DNA sample has great appeal: DNA from a crime scene could be used to create a facial image of a suspect; the faces of prehistoric peoples could be reconstructed from their remains; the face of a child could be predicted in utero from amniocentesis. This is the promise implicit in the study of Claes et al. [1]. In their own words:

“…our methods provide the means of identifying the genes that affect facial shape and for modeling the effects of these genes to generate a predicted face.” (pg. 10)

Unfortunately, this promise greatly overreaches the data and analyses represented by the study, and it misrepresents our current understanding of the genetics of complex morphological traits. Worse, this claim, and the fairly sensational media reports that have stemmed from it, detract significantly from what is otherwise an important paper that highlights the potential of an interesting new technique to investigate the genetic basis for variation in the shape of the human face.

Claes et al. [1] base their analysis on a mixed ancestry sample of 592 people genotyped with a 540,000 single nucleotide polymorphism (SNP) array. The primary analyses conducted deal with the effects of sex and ancestry on facial shape. Sexual dimorphism (when taking ancestry into account) explains 13% of the total shape variation in the face, while ancestry (when taking sex into account) explains 10%. These results are interesting, particularly as the contribution of ancestry is unexpectedly high. This surprising result has tremendous implications for the microevolution of facial shape and, potentially, the role of sexual selection. Could the amount of facial shape variation attributed to ancestry have accumulated through genetic drift? If not, what might explain the surprising contribution of ancestry to facial shape? These questions are intriguing and important regardless of the extent facial shape, or even facial shape characteristics, can be reliably predicted from DNA.

But why is the claim that facial shape can be predicted from DNA so troubling? The first reason is that it isn't actually supported by the work done in this study. Claes et al. [1] examine the effects of candidate genes for normal craniofacial variation. They select 46 genes based on evidence of accelerated evolution and evidence from animal models that these genes are expressed in the head. Here, they argue that, because the genes in question are already known to contribute to head development, no adjustment for multiple comparisons is necessary. This assumption is highly problematic. Lots of genes are involved in the development of the head, but that does not mean that those genes contribute to normal variation in the face. Large-scale genome wide association studies (GWAS) in humans often fail to demonstrate significant roles for genes that are known to produce relevant phenotypes in experimental models. In fact, it is also quite possible that many genes not known to play important roles in craniofacial development contribute to normal variation in the face. Many genes that have known developmental roles may be so important that variation in their function or expression is highly selected against. For example, *Shh* plays crucial roles in forming the face [2], and yet this gene does not appear in GWAS studies of human facial shape variation [3]. Alternatively, variation in a characteristic like facial shape may come from unexpected sources such as genetic variation in gap junction proteins that influence the ability of bone to respond to mechanical load during chewing [4]. Examination of Table S2 reveals that only one of the 46 candidate genes tested would have survived Bonferroni adjustment for multiple testing. Even if one accepts the rationale for avoiding adjustment for multiple testing, the authors could have compared the p values obtained for the 46 genes from random samples of SNPs drawn from the 55,000 tested. In the absence of such a test, the study contributes nothing new to our understanding of how genes influence the shape of the face since the genes tested may or may not actually contribute anything to normal variation in the shape of the face.

**Figure 1:**
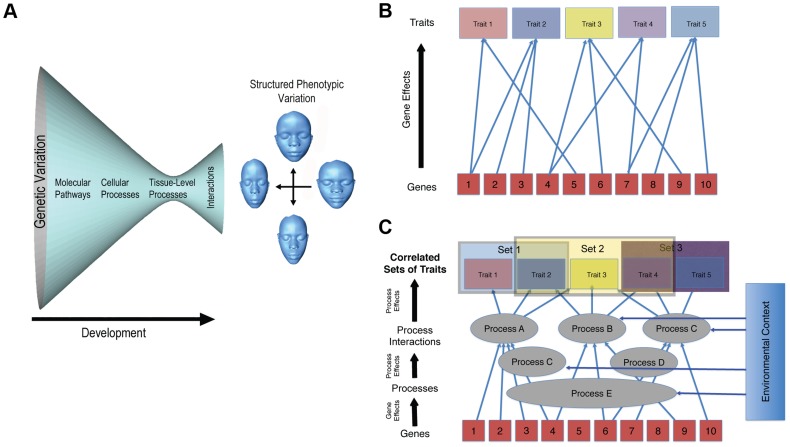
Two complementary depictions of the developmental architecture underlying the genotype–phenotype map for complex traits. A captures the idea that large amounts of genetic variation funnel to smaller numbers of pathways and processes. These processes then interact to produce structured and modulated phenotypic variation in a complex trait. B, which derives from Wagner [20], shows the many-to-many mapping of genes to traits; while both Cs show the modular pattern of gene effects on processes and the effects of processes on sets of phenotypic traits. These depictions illustrate some of the complexity involved in constructing models of genotype–phenotype relations in complex traits.

The second, and deeper, issue, though, is whether genomic prediction of complex morphologies is even feasible. Obviously, variation in genes causes variation at the phenotypic level. This does not mean, though, that a complex phenotype can be accurately predicted from genetic data. For a trait such as coronary heart disease (CHD), prediction of risk is highly problematic, even though quite a lot is known about the underlying genetics [5]. Much less is known about the genetics of complex morphological traits like the shape of the face. The problem is that the genotype–phenotype map for morphological traits is incredibly complicated (Figure 1). It is not just that variation in genes exhibit many relationships with phenotypic outcomes (Figure 1B). It is, rather, that phenotypic variation in morphological traits is structured by developmental processes at multiple levels and times in development. These processes and their interactions are complex but modular in their organization [6], [7]. This architecture of development is responsible for the modulation of phenotypic variance [8] and covariation structure [6], [7] (Figure 1C). Changes to developmental processes that influence the shape of the head tend to produce highly integrated and often unexpected effects on global shape [7], [9]–[11]. Even subtle effects, such as those produced by enhancers to craniofacial genes acting in spatiotemporally specific ways during development, produce global rather than localized shape transformations of the head [12].

Complex patterns of interactions among developmental processes can also generate unexpected patterns of heritability. Although genetic variance may be predominantly additive for complex traits such as oil content in maize or body mass in mice [13], [14], this may not be the case for multidimensional and modularly organized morphological traits like facial shape [15], [16]. If a significant proportion of the genetic variance for facial shape is non-additive, which remains an empirical question, prediction of facial shape from genotype is greatly complicated. The genetic basis for facial shape variation may be as much, if not more, about epistatic interactions and context-specific developmental interactions than about the additive effects of individual genes. The fact that a large, recent GWAS study of facial shape revealed few causative loci is suggestive of a very complex genetic architecture for this trait [3].

The overselling of these results is unfortunate and unnecessary because it detracts from what is otherwise a very interesting study. The bootstrapped response-based imputation modeling (BRIM) technique, for example, is an intriguing extension of the shape analysis toolkit. Like other geometric morphometric methods, it is based on Procrustes superimposition and multivariate data reduction of variation in landmark position. The method allows for estimation of a single quantitative axis of variation that would correspond to a multidimensional factor such as ancestry, or a discrete variable such as sex. The method relies on machine learning algorithms to define shape axes that best correspond to such variables. As such, the method has great potential for furthering quantitative analyses of the genetics of complex morphologies. The use of dense landmark representation and machine learning algorithms similarly has potential in the analysis of complex morphologies, and this study points the way towards future applications of such techniques. This aspect of the paper would be stronger had they validated BRIM by comparison to existing methods. Estimation of the effects of single genes, ancestry, sex or any other factor of interest on total shape variation and local shape variation can already be done using the current GM toolkit [17] or dense correspondence analysis [18], [19]. Although, how much better BRIM performs than existing methods is hard to tell without validation, despite the seemingly encouraging results presented here.

Developing a mechanistic understanding of the genotype–phenotype map is undoubtedly one of the greatest challenges of modern biology. Claes et al. offer us a new and valuable tool to apply to this grand challenge. We should not be fooled, however, into thinking that we are anywhere close to understanding developmental genetics at the level where prediction of complex morphological traits is feasible. Overselling and overpromising in science is dangerous because it creates unreasonable expectations both at the public and policymaker levels. Ultimately, this runs the risk of diverting valuable scientific resources away from the important task of understanding how variation in genes plays through developmental processes to produce the amazing diversity of organismal form.
